# Finding meaning in life while living with HIV: validation of a novel HIV meaningfulness scale among HIV-infected participants living in Tennessee

**DOI:** 10.1186/s40359-015-0070-7

**Published:** 2015-05-02

**Authors:** Carolyn M Audet, Lois J Wagner, Kenneth A Wallston

**Affiliations:** 1 Department of Health Policy, Institute for Global Health, Vanderbilt University Medical Center, 2525 West End Ave Suite 750, Nashville, TN 37203 USA; 2 Nursing and Allied Health, Regents Online Campus Collaborative, Tennessee Board of Regents, Memphis, USA; 3 School of Nursing, Vanderbilt University, 421 Godchaux Hall, Nashville, TN 37240 USA

**Keywords:** HIV Meaning, Psychological well-being, Purpose In life, HIV/AIDS, Southern US

## Abstract

**Background:**

People living with HIV who maintain a positive outlook on their future may manage stress better than those who do not, leading to improved coping behaviors and better health outcomes.

**Methods:**

While studying 125 HIV+ adults participating in two clinical trials of expressive writing we assessed their HIV-specific meaningfulness of life with a short, unidimensional scale (the HIVMS).

**Results:**

The HIVMS had a strong Cronbach’s alpha (0.80) and acceptable test-retest reliability (0.70). HIVMS scores were strongly correlated with measures of perceived control, optimism, and psychological well-being. Participants with lower HIVMS scores had higher probability of non-adherence to antiretroviral medication, suggesting a decreased ability to manage their illness successfully. Neither the control nor expressive writing intervention groups showed increased HIVMS scores.

**Conclusions:**

Future research is necessary to determine the effect of HIV meaning on long-term health outcomes and to develop interventions that can significantly improve a person’s perception of their meaning in life.

## Background

New advances in HIV treatment have effectively made HIV a manageable chronic disease. A 20 year old diagnosed HIV+ between 2006 and 2007 could expect to live an additional 51 years, resulting in a life expectancy of 71 years (Hogg et al. 2013). New antiretroviral medications result in fewer physical side effects and have simplified dosing. Despite advances in treatment, diagnosis with HIV infection is still traumatic. Many people living with HIV experience internalized-stigma related to their HIV status (Berger et al. 2001; Van Rie et al. 2008), stigma and discrimination from family, friends, potential sexual partners, and their communities (Phillips et al. 2011; Loutfy et al. 2012; Fair and Albright 2012; Audet et al. 2013; Lichtenstein et al. 2002), side effects from HIV medications (Panel on Antiretroviral Guidelines for Adults and Adolescents 2013), and increased impact of potential co-infections, including tuberculosis, hepatitis C, and meningitis (Hua et al. 2013). These challenges can impact the psychological and physical well-being of people living with HIV.

Qualitative research has found that people diagnosed with HIV often report a shift in their perspective of the meaning of their lives or purpose after diagnosis (Coward 1994; Farber et al. 2003; Lyon and Younger 2001; Lewis et al. 2006; Schaefer and Coleman 1992; Bower et al. 1998). Reports of increased meaning in life post-diagnosis are common: active community outreach from HIV-support organizations, compassionate family members, caring clinical staff, and psychological support services can help those recently diagnosed to generate and associate positive meaning with their HIV infection (Bower et al. 1998; Lutz et al. 2011; Kremer and Ironson 2014). Others may feel their lives have lost meaning. An HIV diagnosis can limit the formation of intimate partnerships, discrimination or poor health can result in unemployment, and internalized stigma can lead to isolation and depression (Audet et al. 2013; Berger et al. 2001; Bunn et al. 2007).

### The study of meaning-in-life

People have long sought to explore the meaning of human existence (Adler 1958; Frankl 1963; Reker and Cousins 1979; Steger et al. 2006). In an effort to transform seemingly random occurrences into culturally understandable ones, people seek to ascribe meaning to positive (educational attainment, occupational achievement) and negative (disease, accidents, or traumatic incidents) events (Kleinman 1988). Meaningfulness has been defined as “a fundamental sense of meaning, based on an appraisal of one’s life as coherent, significant, directed and belonging” ((Schnell 2009):487). Sources of meaning can include: leisure activities or hobbies, personal relationships, creative work, traditional and culture, legacy, and religion, among others (Reker and Wong 1988). These sources reflect human needs, including “purpose, understanding, responsible action, and enjoyment” (MacDonald et al. 2012). Feeling a strong meaning in life contributes to a healthy sense of coherence (S.O.C.), in which people believe: (1) that events in their lives are explicable and predictable; (2) they possess the resources to cope with difficult events; and (3) these events are worth investing time and energy to successfully manage (Antonovsky 1993). Those with a healthy S.O.C. are hypothesized to manage stress better than those who do not, potentially leading to improved coping behaviors and better health outcomes (Ironson and Kremer 2009; Kremer and Ironson 2014; Giglio et al. 2014; Gison et al. 2014; Geulayov et al. 2014). Being diagnosed with a fatal or serious chronic disease can heighten the need to attribute meaning to a person’s experience and can result in changes in identity, belief in a higher power, or perceived meaningfulness of life (Thorne 1999; Kremer and Ironson 2014; Lutz et al. 2011). If the diagnosis has a negative impact on a patient’s perception about the meaningfulness of their lives, it can be psychologically and physically damaging (Lyon and Younger 2001; Frankl 1963; Bower et al. 2003, Bower et al. 1998).

People who feel their lives have no meaning may cope with the trauma of a serious illness in maladaptaptive ways, including non-adherence to medication regimens and skipping medical appointments. They also may fail to disclose their health status to family and friends, shutting off opportunities for social support (Fife 1995). Previous studies of terminally or chronically ill patients have found associations between belief in the meaning of a person’s life and positive mental health outcomes (Weir et al. 1994; Farber et al. 2003; Fife 1995; Lyon and Younger 2001; Dezutter et al. 2014), self-care activities (Coward 1994), improved physical well-being (Farber et al. 2003; Bower et al. 2003, Bower et al. 1998; Dezutter et al. 2014), life satisfaction (Coward 1994), and mortality (Hill and Turiano 2014).

### Meaning-in-life among people living with HIV

Three quantitative studies using Crumbaugh’s 20 item Purpose in Life (P.I.L.) survey (Crumbaugh 1968) have been conducted with HIV positive individuals in the United States (Bechtel 1994; Lyon and Younger 2001; Lewis et al. 2006). These studies, focused primarily on white, gay men, found that lower perceived purpose in life was more strongly correlated with depression than disease severity (Lyon and Younger 2001) and that HIV positive gay men had lower P.I.L. scores than gay men without HIV (Bechtel 1994). A more recent study found that overall meaning and purpose in life scores among those living with HIV increased over time; however P.I.L. scores among African Americans, those with lower income, and those with only high school education remain low (Lewis et al. 2006). Ironson and Kremer (2009) found psychological well-being and spirituality correlated strongly with the health (viral load, CD4 cell counts) and 5-year survival among people living with HIV.

### Expressive Writing Interventions

Expressive writing therapy was developed by James Pennebaker (Pennebaker 1997) as a therapeutic process for people having difficulty coping with traumatic experiences. People are encouraged to write repeatedly about emotional experiences for at least four consecutive sessions. No feedback is provided by researchers or psychologists; participants are encouraged to reassess, identify, label, and understand distressing experiences through thinking and writing their own narrative (Pennebaker 2010) with the goal of improving psychological and/or physiological health outcomes. This intervention has been used with varying degrees of success with patients diagnosed with rectal cancer (Lepore et al. 2015), renal cell carcinoma (Milbury et al. 2014), Stargardt’s disease (Bryan and Lu 2014), anxiety (Park et al. 2014), depression (Krpan et al. 2013), and HIV disease (Ironson et al. 2013; Westling et al. 2007) among others.

The purpose of this article is to report on the correlates of a short, novel HIV-specific meaningfulness scale among PLHIV who participated in a randomized clinical trial of expressive writing while attending regularly scheduled appointments at an HIV clinic in Nashville, Tennessee. We expected that the patients with high scores on the HIV Meaningfulness Scale (HIVMS) should have greater perceptions of control over their disease and their lives, be more optimistic, have better mental health, and a higher quality of life.

## Methods

### Study procedures

This study was approved by the Vanderbilt University Institutional Review Board (IRB# 031107). Two randomized clinical trials of expressive writing were conducted, but in neither trial was a main effect found for the expressive writing condition. The first trial (Wagner et al. 2010) was a pilot study for the second trial, but the procedures were the same for both trials through the first post-intervention assessment. The results of the second trial have not been published. Baseline data and data from the first post-intervention for these two studies have been pooled for the secondary analyses presented in this paper.

Prior to beginning the study, participants completed the informed consent process. Participants provided information on demographics and completed self-report measures of psychological well-being and psychological coping resources (see below for descriptions of the individual measures). The measures were filled out at baseline in the clinic before participants began the expressive writing study and again 1-month after the fourth and final writing session. Each session lasted 20 minutes, with patients understanding that they would receive no feedback on their written narratives. Sessions were spaced approximately one week apart. The follow-up occurred approximately 7–8 weeks after the baseline assessment.

### Subjects

One hundred and twenty five PLHIV were enrolled in one of two clinical trials examining the effects of expressive writing on positive and negative outcomes related to perceived psychosocial and health status among persons with HIV. These adults had a mean age of 41.7 years, were primarily male (73%), African American (61%) and had an income below $10,000 (71%). All were receiving care for HIV at the Comprehensive Care Center in Nashville, TN. Patients were randomized into either an expressive writing or a control writing condition. See Table 1 (Participant Characteristics at Baseline) for baseline descriptive characteristics of the combined study sample. Our sample was similar to the clinic population, with the exception of race (which was estimated to be 42% African American two years after the study period) (McGowan et al. 2011; Qian et al. 2011).

**Table 1:** **Participant characteristics at baseline**

Characteristic	Mean (SD)	Frequency (%)
Age	41.7 (2.0)	
Male		91 (73)
Heterosexual		62 (49)
Race		
African American		75 (61)
Caucasian		48 (39)
Employment status		
Employed Full/Part Time		31 (25)
On Disability		34 (37)
Annual Income ≤ $10,000		88 (71)
Years with HIV	8.1 (5.6)	
CD4 cell count^1^	432 (338)	
Diagnosed with AIDS		40 (31)
Years with AIDS	2.3 (0.5)	
Sexual Transmission		98 (80)

^1^CD4 cell counts were only available for 75 participants.

### Measures

*HIV Meaningfulness* was measured with four items inspired by the meaning subscale of Antonovsky’s 13-item Sense of Coherence instrument (SOC-13) (Antonovsky 1993). Initially, we engaged experts in scale development and validation, HIV care and treatment, and psychologists working with terminally ill patients to identify appropriate measures. After that review we chose to rewrite questions similar to the S.O.C., but tailored for our population, because it has a well-established internal consistency (alphas of 0.82-0.95 in 16 studies) and has been shown to be stable over time (Antonovsky 1993). We adapted the content for an HIV+ population and reduced the number of items to facilitate administration of the scale to all patients at each clinical encounter. All of the items were worded positively and began with “As a patient with HIV Infection…” (See Table 2 for item wording) and were scored on a 7-point Likert scale. The HIVMS has a Cronbach’s alpha of 0.80, establishing its internal consistency reliability, and correlates 0.62 (n = 125; p < .001) with the 4-item meaning subscale of the SOC-13, establishing its concurrent validity as a measure of “sense of meaningfulness.” An exploratory principal components factor analysis showed that all four of the HIVMS items loaded significantly on a single component that explained 62.5% of the variance. (See Table 2 for factor loadings.)

**Table 2:** **Baseline descriptive data for the HIVMS (n = 125)**

	Mean	SD	Factor loadings
As a patient with HIV Infection…			
1. My life is full of interest.	5.47	1.78	.74
2. When I think about my life I very often feel how good it is to be alive.	5.84	1.81	.78
3. Doing the things I do every day is a source of deep pleasure and satisfaction.	4.94	1.89	.81
4. I anticipate that my personal life in the future will be full of meaning and purpose.	5.51	1.85	.83

SD = standard deviation.

*An Index of Psychological Well-Being* was constructed by subtracting the standardized (z) scores on the Perceived Stress Scale (Cohen et al. 1983) and the Negative Affect subscale of the Positive and Negative Affect Schedule (Watson et al. 1988) from the Positive Affect subscale of the PANAS. The higher the scores on this index, the greater the person’s psychological well-being. This index correlates -.92 (p < .001) with scores on the Center for Epidemiological Studies-Depression scale (Radloff 1977) administered to a subsample of these participants (n = 81), and .77 (p < .001) with the SOC-13 (Mukolo and Wallston 2012).

*HIV-specific Quality of Life* was measured by the total score on the MOS-HIV Scale (Wu et al. 1997). The MOS-HIV was modeled after the SF-36 (Stewart et al. 1988), a well-established measure of health-related quality of life that incorporates subdomains related to both physical and mental health.

The total number of *HIV-related Signs and Symptoms* was assessed by the revised Sign and Symptom Check-List for Persons Living with HIV/AIDS (SSC-HIV), a list of 73 physical signs and symptoms that characterize the condition (Holzemer et al. 2001).

*Dispositional Optimism*, the tendency to look on the bright side of things regardless of the situation, was assessed by the Life Orientation Test (LOT) (Scheier and Carver 1985). *HIV-specific optimism* was measured with a scale modeled after the LOT developed specifically for this study. These two measures of optimistic expectancies correlate .56 (p < .001) at baseline in this sample, providing evidence of the concurrent validity of the HIV-specific measure.

*Generalized Self-Efficacy*, the belief that the self is capable of doing whatever is necessary to bring about desired outcomes, was assessed by the Perceived Competence Scale.(Wallston 2001) *HIV self-efficacy* was measured by the Perceived HIV Self-Management Scale.(Wallston et al. 2011) As reported previously, these two measures of perceived control over one’s behavior correlate .69 (p < .001) at baseline in this sample, providing evidence of the concurrent validity of the PHIVSMS (Wallston et al. 2011).

*Resilient Coping*, the tendency to cope with stressors such as a chronic health condition in a highly adaptive manner, was assessed by the Brief Resilient Coping Scale (Sinclair and Wallston 2004).

An *Index of Adherence to HIV Treatment* was constructed by reviewing patients’ medical records for the 3-month period prior to their enrollment in the study and three months after the forth writing session for indications of failures to keep scheduled appointments and adhere to prescribed medications, if applicable. In addition, patients self-reported their age, sex, race, household income, number of years they have known they were HIV positive, and whether or not they had been diagnosed with AIDS.

### Data analysis

Descriptive statistics for continuous measures are described with the mean and standard deviation. For those subject characteristics that are categorical in nature, percentages for each category were calculated. Spearman’s correlations (rhos) were used to estimate the bivariate associations between the HIVMS and the other psychological measures and demographic variables. Simple change scores from the pre-trial assessment to the 1-month post-writing follow-up assessment were calculated by subtracting the pre-trial score from the post-writing 1-month follow-up score. Dynamic correlations between change scores for the HIVMS and change scores for the other psychological measures were also estimated with Spearman’s rhos, for the total sample and separately for each experimental condition (expressive writing and neutral writing). Paired t-tests were used to see if HIVMS scores changed significantly from pre- to post-writing sessions for the total sample and for each of the two experimental conditions. Significance levels for each test were set at p < 0.05 and no adjustments were made for multiple comparisons.

## Results

Among our participants the HIVMS had a mean score of 5.44 (out of seven) with a standard deviation of 1.45. Table 3 presents Spearman rhos for the baseline associations between HIV meaningfulness and the other psychological attributes assessed. As expected, there are strong positive correlations between HIV meaningfulness and measures of perceived control, optimism, and psychological well-being, and moderate correlations with overall HIV quality of life and resilient coping. These correlations support the convergent validity of the HIVMS.

**Table 3:** **Baseline association of HIVMS with other constructs (n = 124)**

	*rho*
Index of Psychological Well-Being	.60***
HIV Quality of Life	.36***
Dispositional Optimism	.64***
HIV-specific Optimism	.51***
Generalized Self-efficacy	.62***
HIV-specific Self-efficacy	.51***
Resilient Coping	.27*

p < .05; ***p < .001.

As expected, the scores on the HIVMS are uncorrelated with any of the demographic and background characteristics of the study participants, with two exceptions: a weak, but significant, negative correlation with the index of non-adherence to HIV treatment (rho = −.19; p < .05) and a moderate negative correlation (rho = −.30, p = .001) with the number of HIV signs and symptoms.

Table 4 presents the dynamic correlations between *changes* in HIV meaningfulness and *changes* in the other psychological attributes assessed as well as *changes* in HIV symptoms over the roughly two month period between baseline and the one-month post-writing follow-up. All of the dynamic correlations were significant for the combined sample (ps < 0.01, except for generalized self-efficacy where p < 0.05). The moderately strong dynamic correlations for the total sample, however, were mainly due to the correlations of the change scores for the expressive writers (rhos ranged from 0.31 to 0.40), not for those assigned to write on non-emotional topics (rhos ranged from −0.06 to 0.29).

**Table 4:** **Dynamic correlations (rhos) between changes in HIVMS and changes in other constructs for total sample and experimental groups separately**

	Total sample^a^	Expressive writers^b^	Neutral writers^c^
Index of Psychological Well-Being	.29**	.32**	.23
HIV Quality of Life	.27**	.31**	.22
Dispositional Optimism	.30**	.38**	.16
HIV-specific Optimism	.27**	.40**	.00
Generalized Self-efficacy	.19*	.32**	-.06
HIV-specific Self-efficacy	.29**	.32**	.23
Resilient Coping^d^	.33**	.33*	.29

**p < .01; *p < .05; ^a^n = 110; ^b^n = 69; ^c^n = 41; ^d^n = 72, 50, and 22.

We also found that HIV meaningfulness scores did not differentially change over the two-month interval from baseline to the one-month post-writing follow-up (t_(68)_ = 1.46, p = 0.15 for the expressive writers; t_(41)_ = 0.76, p = 0.45 for the neutral writers). As a whole, those asked to express in writing their deepest thoughts and feelings about some stressful or traumatic event, including, perhaps, living with HIV, did not find any more meaning associated with being HIV positive than did those asked to write in a non-emotional manner about some trivial or unimportant topic. In fact, the HIVMS scores for both groups on average decreased two-tenths of a point from baseline (M = 5.52) to the one-month post-writing follow-up (M = 5.33; t_(109)_ = 1.60, p =0.11).

## Discussion

The primary aim of this analysis was to validate a novel HIV meaningfulness scale and explore correlations between this scale with patient adherence and HIV-related symptoms among participants enrolled in treatment at a clinic in Nashville, Tennessee. We also assessed the impact of an expressive writing intervention on HIVMS scores. HIVMS scores suggest the population enrolled in the study felt their lives were meaningful and were optimistic about the future. These scores may reflect the substantial financial and psychosocial support provided by the CCC and Nashville Cares, a local non-governmental organization (Nashville Cares 2014). We expected that patients with higher scores on the HIVMS would have greater perceptions of control over their disease and their lives, be more optimistic, have better mental health, and a higher quality of life. We found significant correlations between HIVMS scores and self-efficacy, coping, optimism, and quality of life, suggesting patients with higher HIV meaningfulness scores may have increased coping abilities which may lead to improved clinical outcomes (Farber et al. 2003; Bower et al. 2003, Bower et al. 1998).

As expected, scores on the HIVMS were not correlated with age, sex, income, work status, ethnicity, or sexual orientation. HIV meaning was negatively correlated with antiretroviral therapy non-adherence and with the number of HIV symptoms, suggesting that people who reported lower levels of meaningfulness in life were less likely to be adherent to their medication and more likely to have symptoms associated with their HIV disease. This association between meaning and adherence and number of HIV symptoms, is similar to that found in other studies (Cederfjall et al. 2002), providing further support for the integration of counseling and other mental health services for patients enrolled in HIV care.

The Cronbach’s alpha of 0.80 for the novel HIVMS in this study shows that this is a reliable (i.e., internally consistent) measure of meaningfulness in a population living with HIV (DeVellis 2003). The strong correlations with the four-item meaning subscale of the S.O.C. and the other psychological measures establishes its construct validity. Further evidence of construct validity is the dynamic correlation between the change in HIVMS and change in HIV optimism, control and well-being among expressive writing participants.

Contrary to our hypothesis, HIVMS scores among both control and intervention participants decreased on average two-tenths of a point at follow-up. In a similar study of women living with HIV, discovery of meaning in life increased among those exposed to an expressive writing intervention, and this increase was associated with increased adherence to medication (Westling et al. 2007). Similar studies on anger management among people suffering from chronic pain (Graham et al. 2008) and lupus or rheumatoid arthritis (Danoff-Burg et al. 2006) have yielded increased scores in life meaning and this increase has been correlated with health outcomes. However, others report that the process of expressive writing yields no increase in meaning-making, particularly among those who already have developed the necessary coping strategies in their everyday lives (Stroebe et al. 2006; Stroebe et al. 2005; Park 2010). Our study, focused primarily on men in treatment, may suggest that that expressive writing does not result in increased meaning in life among this population.

One of the strengths of this study is that, in addition to being able to look at static (i.e., cross-sectional) associations between our measure of HIV meaningfulness and other psychological constructs, we were also able to compute dynamic correlations between the change over time in HIV meaningfulness and change in those other constructs. As shown in Table 4, when looking at the total sample those dynamic correlations were all positive and significant, but, interestingly, the significant dynamic correlations between HIVMS and the other constructs were mostly evident among those in the expressive writing group, not among those assigned to the control writing group. A possible inference to be drawn from this is that some degree of cognitive-emotional restructuring took place among those assigned to do expressive writing that did not occur for those in the neutral writing condition. We can only speculate why the changes in HIVMS scores appeared to track the changes in the other measured psychological constructs for the expressive writers to a greater extent than is evident for the control group writers. One possibility is that the smaller size of the control group relative to the expressive writing group is why some of the correlations in the last column of Table 4 are not statistically significant, but this lack of power doesn’t account for the entire pattern of findings shown in the table. A much more intriguing hypothesis is that the clue to this puzzle lies in the actual narratives written by some of the expressive writers. We are, in fact, pursuing a qualitative analysis of the writings of those assigned to the experimental condition with the idea of linking what we learn from that study back to the changes in the quantitative measures described in this paper.

The results of this study support the use of a novel HIV meaningfulness scale to measure meaning in life among PLHIV. However, in addition to sample size there are several other limitations to acknowledge. In this study we only had pharmacy record data to act as a proxy for medication adherence, and number of HIV symptoms, and length of time since diagnosis was self-reported, potentially leading to misclassification due to social desirability or recall bias. Our population was primarily African American men with low socio-economic status, which may limit our generalizability to the general population. By only including participants that were part of a randomized controlled trial limits our ability to accurately measure test-retest reliability of the measure in the absence of a psychological intervention. Finally, this study did not assess the association of the HIVMS to long-term health outcomes, including morbidity or mortality.

Despite these limitations, using this short HIV-specific meaning scale among patients enrolled in care can have significant implications for health care delivery. Given strong correlations with measures of well-being and resilient coping, clinicians may find this scale allows them to identify patients who need additional psychological or psychosocial support to live positively with HIV. Because of the improvements in HIV treatment, PLHIV can live long and productive lives. The introduction of social support services among a subset of PLHIV who need additional assistance may improve adherence, reduce risky behaviors, and reduce internalized HIV stigma (Farber et al. 2013; Sikkema et al. 2010; Bottonari et al. 2010; Springer et al. 2012).

## Conclusions

HIV meaningfulness is highly correlated with other measures of psychological well-being, namely sense of coherence, measures of control, optimism and well-being. Unlike a similar study with HIV-infected women, our expressive writing intervention was not associated with increasing HIV meaningfulness among our participants. Future research is necessary to determine the effect of HIV meaning on long-term health outcomes, including morbidity and mortality.
